# Multiscale Porosity Microfluidics to Study Bacterial Transport in Heterogeneous Chemical Landscapes

**DOI:** 10.1002/advs.202310121

**Published:** 2024-03-06

**Authors:** M. Mehdi Salek, Francesco Carrara, Jiande Zhou, Roman Stocker, Joaquin Jimenez‐Martinez

**Affiliations:** ^1^ Department of Biological Engineering, School of Engineering Massachusetts Institute of Technology Cambridge MA USA; ^2^ Department of Civil Environmental and Geomatic Engineering Institute of Environmental Engineering ETH Zurich Zurich Switzerland; ^3^ Microsystems Laboratory Institute of Microengineering School of Engineering EPFL Lausanne Switzerland; ^4^ Department of Water Resources and Drinking Water Eawag Dubendorf Switzerland

**Keywords:** chemotaxis, fluid flow, micromodels, microscale porosity, solute transport

## Abstract

Microfluidic models are proving to be powerful systems to study fundamental processes in porous media, due to their ability to replicate topologically complex environments while allowing detailed, quantitative observations at the pore scale. Yet, while porous media such as living tissues, geological substrates, or industrial systems typically display a porosity that spans multiple scales, most microfluidic models to date are limited to a single porosity or a small range of pore sizes. Here, a novel microfluidic system with multiscale porosity is presented. By embedding polyacrylamide (PAAm) hydrogel structures through in‐situ photopolymerization in a landscape of microfabricated polydimethylsiloxane (PDMS) pillars with varying spacing, micromodels with porosity spanning several orders of magnitude, from nanometers to millimeters are created. Experiments conducted at different porosity patterns demonstrate the potential of this approach to characterize fundamental and ubiquitous biological and geochemical transport processes in porous media. Accounting for multiscale porosity allows studies of the resulting heterogeneous fluid flow and concentration fields of transported chemicals, as well as the biological behaviors associated with this heterogeneity, such as bacterial chemotaxis. This approach brings laboratory studies of transport in porous media a step closer to their natural counterparts in the environment, industry, and medicine.

## Graphical abstract

Bacterial chemotaxis under flow conditions in response to a nutrient hotspot (uncaged by photolysis) within a multiscale porosity micromodel.

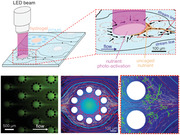

## 1 Introduction

Porous media frequently show a multimodal distribution of pore sizes (void volumes within the total volume), often characterized by both macropore and micropore regions. Multiscale porosity is important in many biological (tissues, blood vessels) and inorganic (soils, rocks) materials, with implications for transport processes in diverse fields including hydrology (soils, aquifers),^[^ ^1^ ^]^ petroleum engineering (oil and gas reservoirs),^[^ ^2^ ^]^ chemical engineering (filters, reactors) ^[^ ^3^ ^]^ bio‐mechanics (bones),^[^ ^4^ ^]^ plant ecology (root–soil interaction),^[^ ^5^ ^]^ and medicine (vascular system, organs, tumors).^[^ ^6^, ^7^, ^8^ ^]^ In many of these applications, the porosity spans several orders of magnitude and can extend down to the micrometer and nanometer scales, such as in bones (with vascular porosity on a scale of 40 µm, down to collagen–hydroxyapatite porosity on a scale of 20 nm) ^[^ ^4^ ^]^ and soils (consisting of sand, pore size 50–2000 µm; silt, pore size 2–50 µm; and clay, pore size < 2 µm). In particular, the porous structure at the smallest scales plays an important role in the behavior of microorganisms in porous environments such as soils,^[^ ^9^ ^]^ for local transport of liquid such as in the cerebral environment,^[^ ^6^ ^]^ and for hydrocarbon recovery in non‐conventional reservoirs, such as shale gas.^[^ ^10^ ^]^

Microfluidics provides a versatile platform to study transport phenomena and the coupling of abiotic and biotic processes in porous media. The approach allows highly precise, quantitative measurements at the pore scale ^[^ ^11^ ^]^ through direct visualization made possible by the use of transparent materials that permit high‐resolution visualization methods (e.g., optical and confocal microscopy), a high level of control of environmental conditions, and high flexibility to rapidly prototype user‐defined geometries. Micromodels can thus be created to mimic fundamental features of real porous environments while allowing monitoring for example of fluid flow, transport processes, and microbial behavior. As a result, microfluidics has already found many applications in research on porous media, including studies of ecology in soils (e.g., bacterial competition, biofilm formation, root growth, fungal development),^[^ ^12^, ^13^, ^14^ ^]^ multiphase flow in soils and reservoirs,^[^ ^15^ ^]^ moisture transport within building materials,^[^ ^16^ ^]^ and transport in organs (e.g., vascular networks, protein‐induced lung inflammation).^[^ ^17^, ^18^, ^19^ ^]^

Most microfluidic models of porous media to date have taken the form of regular or irregular arrays of pillars, with the inter‐pillar spacing, which represents the pore size, typically having a single dominant scale.^[^ ^11^, ^15^ ^]^ More recently, some studies have incorporated greater spatial heterogeneity in microfluidic models to increase realism, by replicating scans of the structure of natural or industrial porous media,^[^ ^20^, ^21^ ^]^ introducing distinct zones with different pore sizes,^[^ ^22^ ^]^ or using a non‐uniform depth of etched features.^[^ ^23^, ^24^ ^]^ Nonetheless, the majority of micromodels have been limited to a single pore size and do not span a wide range of porosities. Furthermore, previous studies were characterized by pore size only down to the micrometer scale, in part due to the technical challenges of fabricating structures with even smaller‐scale porosity.

Experimental observations and mathematical models at different scales suggest that solute transport in porous media is highly dependent on the complexity of the environment. The physical heterogeneity of porous media is largely responsible for strong heterogeneities in the fluid velocity field, which is reflected in non‐Gaussian velocity distributions.^[^ ^25^, ^26^ ^]^ This in turn has consequences for transport processes, leading to anomalous or non‐Fickian transport.^[^ ^27^, ^28^, ^29^, ^30^ ^]^ By creating regions of high and low fluid velocity, the physical heterogeneity controls the residence time of dissolved chemicals and particulates in the porous medium and generates chemical gradients that change over space and time.^[^ ^25^, ^26^, ^27^, ^28^, ^29^ ^]^ These dynamics, in turn, affect fundamental biological processes and shape microbial distribution.^[^ ^31^ ^]^

Beyond the inherent structural complexity of porous materials and its impact on fluid flow and solute transport, there are examples in nature of a further chemical heterogeneity, due to the presence of scattered sources of nutrients, such as carbon from aggregates in natural soils.^[^ ^14^, ^32^, ^33^ ^]^ These sources result in hotspots of resource availability for microorganisms, and when stirred and diluted by the fluid flow, create heterogeneous concentration fields and can form strong gradients. These gradients, together with the fluid flow itself, affect the ability of microorganisms to form colonies such as biofilms,^[^ ^34^ ^]^ as well as their locomotion and chemotaxis (the ability to sense chemical gradients and move toward a source).^[^ ^35^ ^]^ Microfluidic devices with a single pore dominant scale have already enabled the direct observation of bacterial chemotaxis in heterogeneous chemical fields,^[^ ^33^, ^36^, ^37^, ^38^, ^39^, ^40^ ^]^ including ephemeral microscale nutrient patches such as those found in aquatic systems.^[^ ^41^ ^]^ However, the breadth of processes, physical and biological, affected by the structure of porous media, along with the complex interplay between them, highlights the need for more realistic micromodels of porous media.

Here we present a novel approach to fabricate microfluidic models of porous media with pore sizes varying over several orders of magnitude, from nanometers to millimeters. We use a hybrid technology that integrates, through in‐situ photopolymerization, pillars made of polyacrylamide (PAAm) hydrogel, which introduce nanometer‐scale porosity, within a conventional silicone‐based microfluidic system, with pillars that create micrometer‐ to millimeter‐scale porosity. This approach allows the creation of devices with single, double, and triple porosity, where the third level of porosity is contributed by the PAAm hydrogel with nanoscale porosity.^[^ ^42^ ^]^ This new approach enables the study of fluid flow, solute transport, and microbial behavior in geometries that possess more realistic multiscale porosity (e.g., as a conceptualization of soil macroaggregates and microaggregates − the hierarchical, self‐organization of soils).^[^ ^43^ ^]^ As a first application to demonstrate the potential of this novel microfluidic model system, we apply it to questions of ecological and industrial relevance in subsurface environments (soils and aquifers). In particular, we use the release of nutrients from the porous hydrogel pillars in our device to simulate the patchy distribution of nutrients in soils at the microscale, and study two fundamental processes in soils: solute transport and bacterial chemotaxis. Both processes are of great importance in the subsurface, determining the distribution of solutes such as nutrients and pollutants and the ability of bacteria to access them, and thereby mediating fundamental biogeochemical processes such as nitrogen fixation and biodegradation.^[^ ^44^ ^]^ Hydrogel‐integrated systems have previously been used in microbial ecology to study bacterial chemotaxis in unstructured environments.^[^ ^40^, ^45^, ^46^ ^]^ In those earlier studies, hydrogel was used as a porous layer to prevent flow while allowing solute diffusion to generate a concentration gradient, rather than as an element of a porous medium. Here we instead integrate hydrogel pillars as one element of a microfluidic model of a multi‐porosity porous medium.

Using these new micromodels, we first characterize solute transport for porous media of different levels of heterogeneity, comparing single, double, and triple porosity structures. We then demonstrate the application of the micromodels to the study of bacterial chemotaxis.

## 2 Results

### 2.1 Micromodels to Simulate the Structural Heterogeneity of Soils

**Figure 1:**
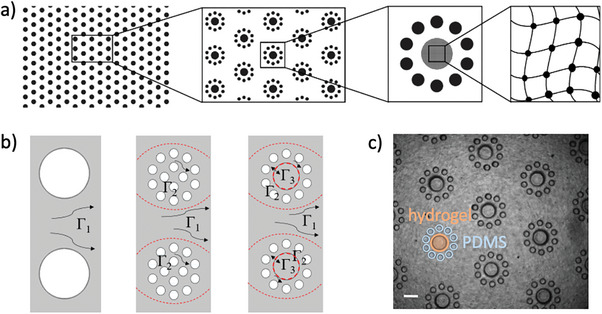
Multiscale porosity micromodels. a) Schematic showing porous domains in the triple‐porosity micromodel. The model is macroscopically homogeneous (left). Successive enlarged sections show pillars made of impermeable PDMS (black) and permeable hydrogel (gray), creating three different scales of pore size ranging from mm to nm. b) Bright‐field image of the triple‐porosity micromodel showing hydrogel and PDMS pillars. Scale bar is 250 µm. c) Single‐ (left) and double‐porosity (center) micromodels were fabricated for comparison with the triple‐porosity micromodel (right). Micromodels differ in their possession of porosity subdomains containing pores of different sizes —subdomain Γ_1_ (between pillar groups), subdomain Γ_2_ (within the groups of smaller pillars), and subdomain Γ_3_ (within hydrogel pillars)—creating transport regions that range from convection dominated (Γ_1_) to diffusion dominated (Γ_3_). Red dashed lines represent the frontier between subdomains. Definitions of pore throat *a* and pore length λ for subdomain Γ_1_ are included in b. Scale bar is 500 µm.

**Figure 2:**
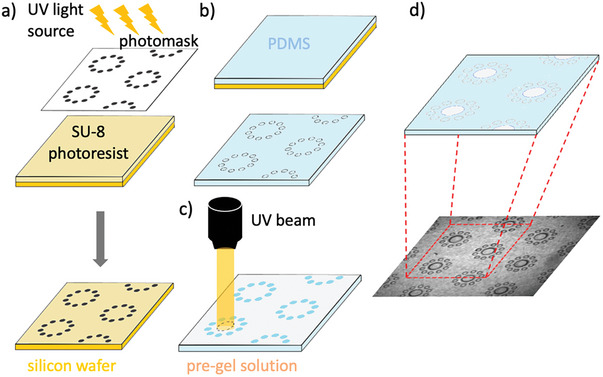
Fabrication of the multiscale porosity micromodel. a) Microstructure molds (yellow) are fabricated by polymerizing SU‐8 photoresist on a silicon wafer by exposure to ultraviolet light through a photomask. b) The array of polydimethylsiloxane (PDMS) pillars is then created by casting PDMS (blue) on the mold. Bonding the structure to a PDMS‐coated glass slide creates a sealed microfluidic channel. c) A pre‐gel solution (gray) is then injected into the microchannel and local photo‐polymerization (curing) occurs upon exposure to UV light to create hydrogel pillars. Unpolymerized pre‐gel solution is washed away using deionized water, leaving an array of PDMS and hydrogel pillars.

The approach relies upon the incorporation of hydrogel pillars that introduce porosity at the nanoscale into a conventional polydimethylsiloxane (PDMS)‐based microfluidic system containing an array of impermeable pillars (**Figure** **1**). The PDMS external walls of the microfluidic channel and the pillar array are fabricated using standard soft lithography techniques.^[^ ^47^, ^48^ ^]^ Thereafter, hydrogel pillars are fabricated by UV photo‐polymerization of a polymer solution within the device in a precise, user‐defined pattern (**Figure** **2**). To demonstrate this technology, we made a triple‐porosity micromodel, with porosity spanning the millimeter to nanometer scale, by fabricating an array of groups of eleven pillars, with each group consisting of ten PDMS pillars of diameter 100 µm surrounding a central hydrogel pillar of diameter 250 µm (Figure 1a,b). This creates three porosity subdomains: Γ_1_ with pore size 1160 µm in the regions between pillar groups, Γ_2_ with pore size ≈116 µm in the regions within pillar groups, and Γ_3_ with pore size <100 nm within the hydrogel pillars ^[^ ^49^ ^]^ (Figure 1c).

For comparison, single‐porosity and double‐porosity micromodels were fabricated using conventional microfabrication techniques in PDMS. The single‐porosity micromodel contained an array of PDMS pillars of diameter 500 µm, creating pores of size 500 µm, of the same order as subdomain Γ_1_ (Figure 1c). The double‐porosity micromodel contained an array of groups of 14 PDMS pillars of diameter 100 µm, forming a heterogeneous porous medium in which the pore size varies over one order of magnitude, creating regions with porosity of the same order as those of subdomains Γ_1_ and Γ_2_ (Figure 1c).

Overall, the three microfluidic systems span a broad range of pore sizes found in natural soils, from nanometers to millimeters, creating a heterogeneous physicochemical landscape that allows the study of transport at the microscale under precisely controlled conditions.

### 2.2 Evolution of Concentration Fields and Breakthrough Curves

**Figure 3:**
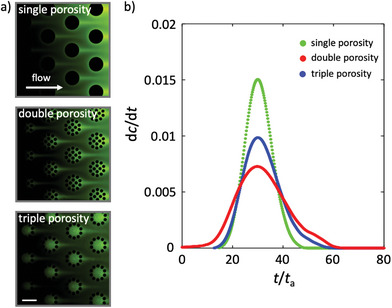
Solute transport through single‐, double‐, and triple‐porosity micromodels. a) Snapshots of the transport experiments (*t*/*t*_a_ > 30) in which deionized water displaces a resident fluorescent (green) solution in each of the porosity micromodels. Scale bar is 500 µm. b) Temporal derivative of the breakthrough curves for the single‐, double‐ and triple‐porosity micromodels. Time on the *x‐*axis was made dimensionless using the advective time *t*_a_ as *t*/*t*_a_. More asymmetric breakthrough curves are observed as the heterogeneity of the porous medium increases, with earlier arrival of the injected deionized water and a longer tailing of the resident fluorescent solution.

The range of porosities of the micromodels (i.e., single, double, or triple porosity) affects the evolution of the solute concentration field and the breakthrough curves (i.e., the time series of the effluent concentration at the outlet) associated with solute transport. To study transport, we filled the three micromodels with a solution of 100 µm fluorescein in deionized water and imaged the reduction in fluorescence over time using epifluorescence microscopy while the channel was exposed to flow of deionized water at a constant flow rate (*Q* = 10 µL min^−1^). To allow comparison of the evolution of solute concentration in the three micromodels (because of differences in mean fluid flow velocities due to differences in total porosity), time *t* was made dimensionless using the advective time *t*_a_, as *t*/*t*_a_ (see Experimental Section). Our observations show that, at early times (*t*/*t*_a_ < 30), the invasion of the porous medium by the injected deionized water displacing the resident fluorescent solution was different for the different micromodels. At later times (*t*/*t*_a_ > 30), concentration fields for the three micromodels show plumes of high solute concentration lingering downstream of the pillars amidst the displacing deionized water flow (**Figure** **3**a). These plumes were more sustained in time for the triple‐porosity micromodel, because the smaller porosity of the hydrogel pillars and through which the main transport process is diffusion.

A measure of the impact of the spatial structure of the porous medium on solute transport is given by a comparison of the breakthrough curves. Breakthrough curves, widely used to describe solute transport in subsurface research, provide a measure of the time‐resolved concentration *c*(*t*) of the solute under investigation at a given position in the system, often the outlet. They can also be interpreted as the residence time distribution, with the portions breaking through later corresponding to longer residence times in the system. To compare the micromodels, we computed the time derivative of the breakthrough curves in each micromodel (d*c*/d*t*; Figure 3b). We found that micromodels with greater heterogeneity were characterized by earlier breakthrough and longer tailing, i.e. there was both earlier arrival of the deionized water at the outlet and a longer tail of residence times of the fluorescent solution. The time derivative of the breakthrough curve was more non‐Gaussian and asymmetric with increasing heterogeneity, i.e., more right‐skewed in the triple‐ than in the double‐ and in the single‐porosity micromodels (Figure 3b). As we show below, these observations can be explained by the heterogeneity in the fluid flow velocity field within the porous medium.

### 2.3 Velocity Field

The velocity field within the micromodels showed a greater velocity contrast between low and high velocity regions as the degree of heterogeneity in pore sizes increased. To characterize fluid flow dynamics, we performed numerical simulations using COMSOL Multiphysics (COMSOL, Burlington, MA) to obtain the full velocity field over the entire micromodels, including in the hydrogel pillars within the triple‐porosity micromodel. The numerical model used a standard hybrid formulation, the Brinkman equation,^[^ ^50^ ^]^ where flow through the larger pores is described by the Navier–Stokes equations and flow through the hydrogel pillars by the Darcy equation. The Reynolds number was much smaller than 1 in all subdomains of all micromodels (**Table** **1**), confirming that the fluid flow was creeping flow (i.e., Stokes flow).

**Table 1:** Control parameters for the different micromodels. Columns show the porosity (*ϕ*) and mean fluid flow velocity ($\bar{u}$), and values for each subdomain Γ*_i_* for the pore throat (*a_i_*), pore size (λ*_i_*), mean fluid flow velocity (*u_i_*), characteristic velocity (local mode) (*u_ci_*), Reynolds (*Re_i_*) and Peclet (*Pe_i_*) numbers.

Micromodel	*ϕ*	$\bar{u}$ (µm s^‐1^)	Γ*_i_*	*a_i_* (µm)	λ*_i_* (µm)	*u_i_* (µm s^‐1^)	*u_ci_* (µm s^‐1^)	*Re_i_*	*Pe_i_*
Single porosity	0.78	98.3	Γ_1_	500	1160	98	95	5.15 × 10^−3^	23.6
Double porosity	0.88	88.5	Γ_1_	500	1160	144	107	8.06 × 10^−3^	34.5
			Γ_2_	50	116	26.6	24.4	1.49 × 10^−4^	0.6
Triple porosity	0.91	89.6	Γ_1_	500	1160	145	115	8.11 × 10^−3^	34.7
			Γ_2_	50	116	3.86	2.96	2.16× 10^−5^	0.09
			Γ_3_ ^a)^	–	–	2.58 × 10^−3^	2.70 × 10^−3^	8.66 × 10^−8^	8.6 × 10^−4^

^a)^Within the hydrogel (subdomain Γ_3_), pore throat and pore length were assumed to be equal for the calculation of *Re* and *Pe*.

**Figure 4:**
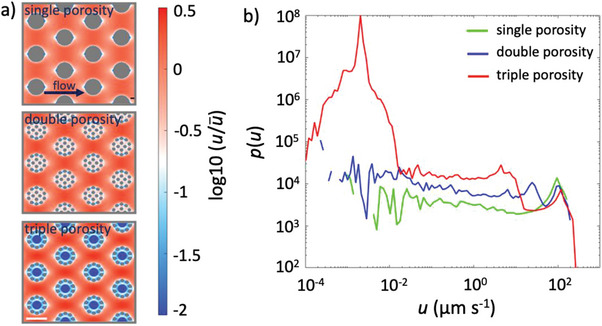
Velocity field in the single‐, double‐, and triple‐porosity micromodels. a) Simulated velocity field in the micromodels (single, double, and triple from top to bottom). The velocity fields were normalized and plotted on a logarithmic scale (color scale) to highlight the velocity contrast between domains. The scale bar is 500 µm. b) Velocity distribution within single‐, double‐ and triple‐porosity micromodels. Double‐ and triple‐porosity micromodels show additional peaks at lower velocities (one and two additional peaks in double and triple porosity, respectively), corresponding to the additional porous subdomains.

Velocity contrasts in a porous medium arise due to the formation of both preferential channels of high fluid flow velocities and zones of low fluid flow velocity, leading to a so‐called double structure of the flow field.^[^ ^51^ ^]^ The fraction of the pore space acting as preferential channels (red tones, **Figure** **4**a) and low‐velocity zones (white and blue tones, Figure 4a) increased, resulting in a more heterogeneous velocity field, as heterogeneity increased.

The distribution of fluid flow velocities varied with the heterogeneity of the micromodels (Figure 4b). The probability density function of the Eulerian velocity magnitude *u*, *p*(*u*), becomes broader with the inclusion of porosities at different scales, i.e. the probability of both high and low velocities increases as physical heterogeneity increases. The distributions show a peak at high velocity before falling off exponentially, with a slight shift of the peak toward higher velocities (i.e., an increase in the characteristic velocity) as heterogeneity increases with the inclusion of regions of lower porosity. Double‐ and triple‐porosity micromodels show additional peaks at lower velocity, with one and two peaks, respectively, corresponding to the additional porosity subdomains (Γ_2_ and Γ_3_) (Table 1).

The transport regime within each of the porosity subdomains (Γ_1_, Γ_2_, and Γ_3_) can be characterized in terms of the Peclet number, *Pe*, which provides a measure of the relative importance of transport by advection and by diffusion (see Experimental Section). Using the peaks in *p*(*u*) to determine the most probable fluid flow velocity within each subdomain (Figure 4b), we confirmed the expectation that the value of *Pe* increases with pore size, so that *Pe*_Γ3_ < *Pe*_Γ2_ < *Pe*_Γ1_. Values of *Pe* indicate that transport within Γ_3_ is dominated by diffusion (*Pe*_Γ3_ << 1), transport within Γ_1_ is dominated by advection (*Pe*_Γ1_ > 1), and the contribution of diffusion and advection is comparable within Γ_2_ (*Pe*_Γ2_ ≈1) (Table 1).

### 2.4 Interplay of Bacterial Chemotaxis and Hydrodynamics in a Triple‐Porosity Micromodel

The multiscale structure of the triple‐porosity microfluidic model, mimicking the spatially heterogeneous environments experienced by many bacteria, provides a new means to investigate the interplay between hydrodynamics and bacterial motility in porous media. Specifically, the inclusion of hydrogel pillars within the triple‐porosity micromodel allows the study of chemotaxis (i.e., motility directed along a chemical gradient) of bacteria toward localized nutrient sources. In such a system, bacterial behavior is directly coupled with fluid flow. In subdomain in Γ_1_, the average flow velocity (*u*_1_ ≈ 145 µm s^−1^; Table 1) was higher than the mean swimming speed of the bacteria we studied, *Vibrio ordalii* (*v* = 46.5 µm s^−1^) (see Experimental Section): in this subdomain bacteria will thus be subject to strong advection by the flow. Within subdomain Γ_3_ the average velocity is very low (*u*_3_ ≈ 0.003 µm s^−1^; Table 1), but the small pore size does not permit bacteria to access this region. Within subdomain Γ_2_, the average flow velocity (*u*_2_ ≈ 4 µm s^−1^; Table 1; Figure S1a, Supporting Information) is lower than the average swimming speed of bacteria, and comparable to the chemotactic velocity of *Vibrio ordalii*:^[^ ^52^ ^]^ we thus expect chemotaxis to be effective within this domain.

**Figure 5:**
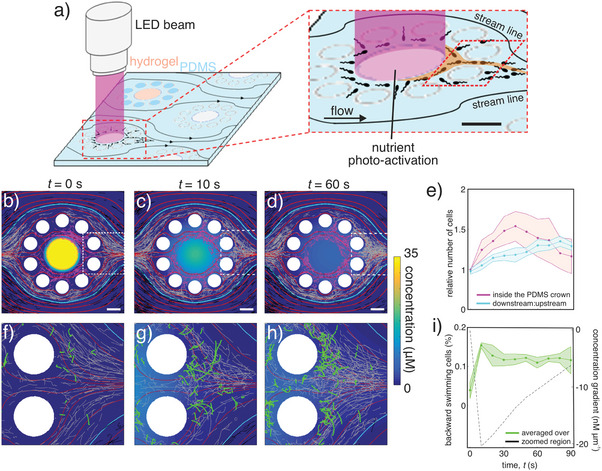
Microbial chemotaxis under flow conditions in response to a nutrient hotspot within the triple‐porosity micromodel. a) Schematic of photolysis. Exposure to an LED beam uncages glutamate, making a chemoattractant pulse almost instantly available to bacteria.^[^ ^51^ ^]^ There is constant flow through the micromodel of a solution containing caged glutamate and bacteria. Scale bar is 100 µm. b–d) Bacterial trajectories over a 10 s period (color coded according to the local flow rate) of a population of *Vibrio ordalii*, superimposed over the streamlines (red) and the nutrient concentration field (color coded). Panels show the periods a) *t* = −10–0 s, b) *t* = 0–10 s, and c) *t* = 50–60 s after the nutrient began to diffuse out of the central hydrogel pillar (yellow circle in b). The glutamate nutrient pulse (35 µM) generated by photolysis had initial cross‐section equivalent to the central hydrogel pillar (250 µm). Cyan lines indicate the boundary where fluid velocity *u* = 100 µm s^−1^. Bacterial trajectories are color coded according to the local flow rate: high flow (*u* > 100 µm s^−1^, black), medium flow (10 < *u* < 100 µm s^−1^, gray), and low flow (*u* < 10 µm s^−1^, magenta). Magenta trajectories correspond to the region within the PDMS crown (subdomain Γ_2_). Scale bar is 100 µm. e) Following the chemoattractant pulse, the concentration of bacteria increased within the central region and downstream of the pulse. Magenta curve: number of bacteria over time within the PDMS crown relative to the number of bacteria at time *t* = 0 s. Cyan curve: number of bacteria downstream (right side) relative to the number of bacteria upstream (left side) within the medium‐ and low‐flow regions (*u* < 100 µm s^−1^; within the cyan lines in panels b–d). Shaded regions show the s.e. of three replicates. f–h) Enlarged views of the areas delimited by the dashed white boxes in panels b–d. Green and gray trajectories show bacteria swimming upstream and downstream, respectively. i) Percentage of bacterial trajectories directed upstream over time within the magnified region shown in panels f–h (green curve; shaded region is ±  s.d around the mean of three replicates). The dashed black curve shows the spatial average of the concentration gradient over the same region (see Figure S2, Supporting Information for examples of the spatial map of the concentration gradient at *t* = 10 s and *t* = 60 s).

To create localized nutrient sources in a non‐invasive manner, we used a caged chemoattractant, 4‐methoxy‐7‐nitroindolinyl‐(MNI)‐caged‐L‐glutamate. Note that similar compounds, such as poly‐γ‐glutamic acid (γ‐PGA), are also responsible for the soil aggregates' stability.^[^ ^53^ ^]^ MNI‐caged glutamate is a version of the amino acid glutamate – a chemoattractant for many bacteria – that is made undetectable to bacteria by caging, a chemical modification with a protecting group that can be removed by exposure to a UV beam. To create controlled nutrient pulses that mimic those that bacteria can encounter in porous media, for example, due to lysis of larger organisms or transport of solutes by flow, we used photolysis within the triple‐porosity micromodel to locally release known amounts of glutamate within the chemically dilute background (**Figure** **5**a). We first filled the device with a solution containing MNI‐caged glutamate, then started to flow into the micromodel a suspension of *Vibrio ordalii*. We created a pulse of glutamate by exposing the volume occupied by one hydrogel pillar to a focused, 500 ms LED pulse ^[^ ^52^ ^]^ and 250 µm diameter: this released a pulse of glutamate corresponding to a local initial concentration of uncaged glutamate of *C*_0_ = 35 µM, for a total amount of glutamate released of 0.11 pmol and a maximum gradient of 20 nM µm^−1^ (see Experimental Section and [52]). Following photolytic uncaging, glutamate diffuses out of the hydrogel pillar and is deformed into a plume downstream of the pillar by the fluid flow. Because glutamate is a chemoattractant for *V. ordalii*,^[^ ^52^ ^]^ the plume induces chemotaxis of the bacteria, which we measured using phase‐contrast microscopy followed by image analysis to reconstruct bacterial trajectories.

The chemotactic response of bacteria to the chemoattractant pulse was strongly influenced by the regions of different porosity, and hence fluid velocity. A comparison of the trajectories of bacteria before (Figure 5b) and after the release of chemoattractant (Figure 5c,d) showed that bacteria responded rapidly (within 10 s from the release) to the high concentration gradients of glutamate diffusing from the pillar, but also that their chemotactic response was significantly affected by the fluid velocity field. Within subdomain Γ_1_, bacteria were mainly advected by the fluid flow, so that regardless of the chemoattractant pulse, the trajectories essentially followed the streamlines (Figure 5b–d; Figure S2, Supporting Information; black trajectories). The trajectories of bacteria in subdomain Γ_2_ and in the region downstream of the pillars included bacteria swimming upstream (Figure 5f–h; green trajectories) because of both shear‐induced reorientation owing to their elongated shape ^[^ ^54^ ^]^ (Figure S1b, Supporting Information) and chemotaxis up the nutrient gradient (Figure S1c,d, Supporting Information). This led to a progressive accumulation of bacteria within the Γ_2_ subdomain, which peaked 40 s after the nutrient release, and in the region downstream of the pillar (Figure 5e). The bacterial chemotactic response, quantified by the relative frequency of bacteria swimming upstream toward the pillar within the region downstream of the pillar, peaked at *t* = 10 s corresponding to the peak time of the concentration gradient of glutamate, but remained at a plateau over the full 90 s of the observations while the concentration gradients gradually dissipated over time (Figure 5i; Figure S1c,d, Supporting Information).

In order to investigate the role of the hierarchical organization within the porosity micromodels on the bacterial response, bacterial chemotaxis experiments have been carried out in double‐porosity micromodels (i.e., without the PDMS crown, subdomain Γ_2_): i) hydrogel pillars of 250 µm diameter, and ii) hydrogel pillars 390 µm diameter (Figure S3, Supporting Information). We found that the bacterial accumulation around the central hydrogel pillar in both the double‐porosity micromodels was strongly reduced irrespective of the size of the hydrogel pillar (Figure S4a,c, Supporting Information) when compared to the triple‐porosity micromodel, at comparable mean fluid flow velocity (Figure S4b,d, Supporting Information) and equivalent initially released glutamate concentrations. The greater retention of bacteria sustained over longer times observed in the triple‐porosity micromodel is due to a combination of lower fluid flow velocities within the subdomain Γ_2_ and higher chemoattractant concentrations sustained over time in the region downstream of the hydrogel pillar.

## 3 Discussion

The versatile porous structures that can be generated by combining hydrogel synthesis and traditional PDMS microfabrication open a wide range of possibilities in research fields that can benefit from more realistic models of porous media, such as soil ecology, subsurface engineering, and medicine.^[^ ^11^, ^14^ ^]^ The inclusion of hydrogel structures allows the incorporation of regions with porosity down to the nanometer scale,^[^ ^42^ ^]^ enabling the engineering of porous media with porosity that spans four to five orders of magnitude. The hydrogel also allows for the release of chemicals by diffusion, so that hydrogel structures can be used directly as a model of point sources of solute.

The hydrogel‐PDMS integrated system offers the possibility to study fluid flow and solute transport processes over a wide range of pore sizes relevant to natural systems. Here we demonstrated its use by comparing breakthrough curves within micromodels with single, double, and triple porosity. Because solute transport experiments were run under the same conditions (i.e., flow rate), the observed differences in the solute breakthrough curves (first arrival and tail) were the result of the different heterogeneity of the three systems, i.e. the inclusion of porosity at different scales. In the triple‐porosity micromodel, the variation in porosity led to the coexistence of highly channelized flow within the largest pores and low‐velocity zones within the hydrogel. Open questions remain in this respect, including for example the understanding of the flow mechanisms (e.g., slippage) in such nanoporous media.^[^ ^42^, ^49^ ^]^ Additionally, the micromodel is still only an approximation of real systems, because it covers a discrete rather than continuous range of porosities, where the low velocities in the velocity distribution usually drop‐off algebraically following a power‐law.^[^ ^55^, ^56^ ^]^

In heterogenous systems, the broad distribution of velocities typically leads to a superdiffusive spreading of solutes.^[^ ^57^ ^]^ The inclusion of very low porosity subdomains such as hydrogel pillars increases the residence time of a solute, as advection and dispersion is greatly reduced locally and the main transport process is diffusion. The sustained solute plumes observed in the triple‐porosity micromodel downstream of the hydrogel pillars also lead to a significant increase of the surface available for fluid mixing.^[^ ^58^ ^]^ The enhancement of mixing resulting from the increased interface between resident and invading solutions is analogous to observations made at continuum scale (i.e., Darcy scale, averaging over thousands or millions of pores) due to heterogeneous flow topologies resulting from a heterogeneous permeability field.^[^ ^59^, ^60^ ^]^ Concentration gradients along the solute plume downstream of the hydrogel pillars are mainly developed in the direction transverse to the mean flow direction, and it is here that the highest reaction rates are expected to occur. In natural porous media such as soils, this spatial structure of concentration gradients is expected to significantly impact the upscaled chemical reactions ^[^ ^61^ ^]^ as well as reactions driven by chemotactic bacteria that navigate in response to such gradients.^[^ ^35^, ^39^, ^62^ ^]^

Under flow conditions, the spatial distribution of chemotactic bacteria is the result of an interplay between fluid dynamics, concentration fields, and swimming behavior.^[^ ^63^ ^]^ The micromodel proposed here offers the possibility to study systematically how these interactions affect the spatial distribution and residence time of bacteria in porous media. Within such systems, the average fluid velocity of each subdomain mainly scales with the local length scale. Within our micromodels, in the Γ_1_ subdomain the average fluid velocity is higher than the swimming speed of bacteria, resembling the situation in soil macropores. This creates a region of high *Pe*, and thus solute transport is mainly controlled by advection. Given that bacterial swimming speed is lower than the velocity of the medium, bacteria traveling through this subdomain are to a good approximation simply being transported by the flow. In the Γ_2_ subdomain, the average fluid velocity is lower than the characteristic swimming speed of bacteria, and in this region of intermediate Peclet number the contribution of diffusion to solute transport is comparable to that of advection. In this subdomain, bacteria can swim against the flow and are exposed to nutrients diffusing from the point sources and advected downstream. The Γ_3_ subdomain is a diffusion‐dominant area, characterized by a low Peclet number. Microorganisms cannot enter this region because of the nanometer pore scale,^[^ ^42^ ^]^ but can accumulate in the surroundings of the source of nutrients and possibly attach to the surface. These processes represent close mimics of the processes of bacterial navigation and recruitment in patchy environments with nanoporosity such as soil aggregates.^[^ ^43^, ^44^, ^64^ ^]^

Over the longer term, the spatial heterogeneity of nutrients and flow dynamics control the population dynamics of bacteria by determining the timescale and nature of interactions, such as cooperation and competition between species.^[^ ^65^, ^66^ ^]^ Hydrogel‐integrated micromodels allow precise control over the rate and duration of the release of nutrients within the porous medium by tuning the permeability of the pillars and by using spatially and temporally controlled photolysis within a constant flow of caged nutrients, thereby enabling long‐term experiments. Under dynamic conditions, the interaction between flow, chemical gradients, and chemotaxis determines bacterial growth and the formation of microbial colonies.^[^ ^67^, ^68^, ^69^ ^]^ Bacterial growth and the development of biofilms (surface‐attached microbial communities within an extracellular matrix) are generally studied with homogeneous (i.e., well‐mixed) nutrients and physico‐chemical conditions (e.g., pH and redox). More recent work demonstrates the interplay between hydrodynamics and the formation of biofilms in porous media, whereby bacterial growth leads to redirection of the flow,^[^ ^34^ ^]^ which in turn controls accessibility to nutrients. This new device will allow the study of such dynamic coupling of flow, heterogenous chemical landscapes, and microbial ecology in a systematic way.

## 4 Conclusion

Microfluidic devices represent a powerful tool to study the interactions of fluid flow, solute transport, and bacterial processes such as chemotaxis, growth, and community development, offering the ability to precisely control fluid flow and mimic natural microenvironmental conditions, while allowing optical access to observe and quantify the interactions. The application of multiscale hybrid micromodels could significantly improve our understanding of fluid flow and transport processes in natural porous media (e.g., tissues, blood vessels, organs, natural soils, rocks, and aquifers), and thereby contribute to diverse applications such as drug delivery, soil bio‐remediation or soil consolidation by bio‐mineralization. For subsurface applications in particular, the ability to create more realistic model systems should catalyze a better understanding of the spatial distributions and residence times of bacteria in heterogeneous concentration fields, thereby promising to improve our predictions of reactive phenomena.

## 5 Experimental Section

### Fabrication Of Silicone‐Based Microfluidic Systems

Microfluidic devices with an array of pillars creating single‐ or double‐porosity structures (Figure 2) were fabricated out of polydimethylsiloxane (PDMS) using soft lithography techniques.^[^ ^47^, ^48^ ^]^ The pillars of the porous domains were designed using computer aided design software and printed onto a transparent film to create a photomask. Microchannel molds were fabricated by depositing and spin‐coating SU‐8 photoresist (Sigma‐Aldrich) on a 10‐cm‐diameter silicon wafer, positioning the photomask on the silicon wafer, then exposing the photoresist to ultraviolet (UV) light to polymerize the regions of interest, and the rest will become the voids in the micromodel itself. Microfluidic channels were created by casting PDMS (Sylgard 184 Silicone Elastomer Kit, Dow Corning, Midland, MI) onto the molds, and after removal, inlet and outlet ports were punched to provide access for tubing. PDMS channels were sealed by bonding to a glass slide using a plasma treatment and heating at 80 °C for 1 h.

### Integrating Nanoscale Porosity to Create a Domain with Triple Porosity

The triple porosity microfluidic device was fabricated in a two‐step process out of two transparent materials (Figure 2): i) PDMS, impermeable to liquids, to form the external walls of the channel and an array of pillars as described above, and ii) hydrogel, permeable to fluids and solutes,^[^ ^70^ ^]^ to incorporate a second type of pillar with nanometer‐scale porosity. In the first step, the base‐channel and pillars of the microfluidic device were fabricated in PDMS using soft lithography as described above. The PDMS channel was then sealed with a layer of PDMS that had previously been bonded to a glass slide using a plasma treatment and heating at 80 °C for 1 h. Sealing with PDMS, rather than with just a glass slide, is necessary to ensure attachment at both the top and the bottom of the hydrogel pillars added in the second step.^[^ ^71^, ^72^ ^]^ Before creating the hydrogel structures, PDMS surfaces were treated with 10% (wt./vol.) benzophenone solution in ethanol for 10 min and then washed with methanol and dried with nitrogen to clean the surfaces and promote the bonding.

In the second fabrication step, the nanopore hydrogel pillars were created. As a hydrogel, we used a polymer solution composed of 10 mL 20 wt.% acrylamide and 1 wt.% N,N–methylenebisacrylamide plus 200 mg Irgacure.^[^ ^71^, ^72^ ^]^ Note that the final hydrogel porosity (and therefore permeability) can be tuned by changing the concentration of the polymer solution.^[^ ^73^, ^74^, ^75^ ^]^ The microfluidic channel was filled with the hydrogel polymer solution, and then the gel was cured at the desired locations of the pillars by UV photo‐polymerization. Polymerization to form the pillars was achieved by exposing the polymer solution to a UV beam (350–360 nm) using a microscope, with the location of each pillar controlled by the microscope translation stage and the diameter controlled by the aperture of the diaphragm. Deionized water was then used to wash out the unpolymerized pre‐gel solution.

### Solute Transport Experiments

The impact of physical heterogeneity on solute transport was characterized for the single‐, double‐, and triple‐porosity microfluidic models. The microchannels were first filled with a solution of 100 µm fluorescein in deionized water. Then quantified the reduction in fluorescence over time as the channel was exposed to flow of deionized water, using a syringe pump (Harvard Apparatus) at a constant flow rate (*Q* = 10 µL min^−1^). Images of the micromodels were recorded every 1 s during 30 min with an epifluorescence microscope (Nikon Eclipse Ti‐E) equipped with a CCD camera (Hamamatsu) with 4 × objective (numerical aperture = 0.2), giving a resolution of 6.5 µm pixel^−1^. At each time point and for each pixel, the reduction in fluorescein concentration is quantified, *c* = 1 − *C*/*C*_b_, where *C* and *C*_b_ are the measured fluorescent intensity and the initial fluorescent intensity of that pixel, respectively.^[^ ^76^ ^]^ The images of the temporal evolution of the fluorescein concentration field were used to compute the breakthrough curves (measuring the solute concentration over time at a fixed position, closest to the outlet) of the porous domain for each micromodel. The Péclet number was used to characterize the relative importance of advective versus diffusive transport within each porous subdomain (Γ_1_, Γ_2_, Γ_3_) (Figure 1b, Table 1). In our geometries, the Péclet number for each subdomain was computed as the ratio of the characteristic diffusion time to the characteristic advection time, *Pe*  = *t*_d_/*t*_a_ , where the characteristic diffusion time over the pore throat *a* (shortest distance between two pillars) is *t*_d_ = *a* ^2^ /(2*D*) with *D =* 450 µm^2^ s^−1^ the diffusion coefficient of fluorescein in water,^[^ ^77^ ^]^ and the characteristic advection time over a pore size *λ* (largest distance between two pillars) is *t*_a_ =  λ/*u* with *u* the mean fluid flow velocity in the subdomain (Γ*_i_*). This yields *Pe*  = *t*_d_/*t*_a_  =  *ua* ^2^/(2*D*λ).

### Fluid Flow Simulations

Fluid flow dynamics were determined from numerical simulations using COMSOL Multiphysics® to obtain the full velocity field, including in the nanoporous regions. The domain's dimensions are *L* × *W* × *h*  =  12.5 mm ×  24 mm ×  0.195 mm. The bulk porosity *ϕ* of each micromodel design is provided in Table 1. The numerical model used a standard hybrid formulation, the Brinkman equation,^[^ ^50^ ^]^ where the flow through the larger pores is described by the Navier–Stokes equations and the flow through the permeable obstacles by the Darcy equation (permeability, κ = 10^−13^ m^2^). A 2D model was adopted, representing the mid‐plane of the experimental micromodels, and the impact on the velocity field of the third dimension (i.e., the no‐slip conditions at the upper and lower boundaries of the experimental domain) was modeled by introducing a Darcy‐like term to account for the drag force exerted by the upper and lower walls.^[^ ^78^ ^]^ No‐slip conditions were defined for the remaining liquid–solid interfaces. The flow was characterized in the different subdomains (Γ_1_, Γ_2_, Γ_3_) by the Reynolds number (Table 1), the ratio of inertial forces to viscous forces, *Re*  =  ρ*ua*/μ, where *ρ* (997.05 kg m^−3^) and *µ* (8.91 × 10^−3^ kg m^−1^ s^−1^) are the density and the dynamic viscosity of the liquid, respectively. The flow was laminar under all conditions tested.

### Bacterial Chemotaxis Experiments

To demonstrate the potential of our triple‐porosity device in the study of bacterial behavior in realistic microenvironments containing microscale point sources within complex porous media, we carried out experiments to visualize the chemotaxis of *Vibrio ordalii* 12B09 toward the amino acid glutamate. *V. ordalii* was grown overnight for 20 h in 2216 medium on an orbital shaker (600 rpm) at 30 °C. The cells in late exponential phase (OD_600_ ≈ 1) were harvested, washed them with filtered autoclaved artificial water (salinity = 36 g kg^−1^), and gently resuspended them for use in experiments to a final concentration of 5 × 10^7^ cells mL^−1^ (at low concentration to allow the tracking of individual cells) in in the same water containing 1 mm 4‐methoxy‐7‐nitroindolinyl‐caged‐L‐glutamate (MNI‐caged glutamate; Tocris Bioscience). The MNI‐caged glutamate is a version of the amino acid glutamate – a chemoattractant for many bacteria – that is made undetectable to bacteria by caging, chemical modification with a photo‐removable protecting group. Prior to the chemotaxis experiments, the triple‐porosity microfluidic model was filled with 1 mm MNI‐caged glutamate in artificial water to allow it to diffuse into the hydrogel pillars for 20 min. During experiments, the dilute bacterial suspension (also containing MNI‐caged glutamate) was injected at constant flow rate (10 µL min^−1^) using a syringe pump (Harvard Apparatus PHD). The mean swimming speed of *V. ordalii* (46.5 µm s^−1^) obtained by tracking single cells under still conditions (as described in [52]) was lower than the mean fluid flow velocity within the porous subdomain Γ_1_ (121 µm s^−1^) but higher than that in subdomain Γ_2_ (10 µm s^−1^, Table 1). In the Γ_2_ subdomain with fluid flow velocities lower than bacterial swimming speed, the migration of bacterial cells toward the chemoattractant was monitored.

To create controlled, dynamic nutrient pulses that mimic those that bacteria might encounter in porous media, photolysis was employed to introduce and make almost instantly available to bacteria the amino acid glutamate with known concentration within a chemically dilute background.^[^ ^52^ ^]^ By exposing the volume occupied by one hydrogel pillar to a focused LED beam (wavelength 395 nm) of the same diameter as that of the pillar, a controlled quantity of glutamate is photoreleased (“uncaged” by photolysis) from the MNI‐caged glutamate in a vertical column. The amount of glutamate was controlled that was uncaged by tuning the exposure time of the LED pulse, as the two quantities are linearly related.^[^ ^52^ ^]^ Application of the focused LED beam for a duration of 500 ms initiates rapid uncaging of the MNI‐caged glutamate.^[^ ^79^ ^]^ An LED pulse of 500 ms corresponds to a concentration of uncaged glutamate of *C*_0_ = 35 µm, obtained through a calibration scheme previously described.^[^ ^52^ ^]^ In the experiments, considering the dimensions of the hydrogel pillar (diameter: 250 µm, height: 195 µm), this is equivalent to a pulse of 0.11 pmol. Following uncaging, glutamate diffuses out of the hydrogel pillar, triggering bacterial chemotaxis. The nutrient was retained by the hydrogel due to its low permeability (κ = 10^−13^ m^2^) and exits the pillar mainly by diffusion,^[^ ^80^ ^]^ creating a gradient around the hydrogel pillar that was deformed into a plume oriented downstream by advection from the surrounding flow (Figure 3a, Figure S1, Supporting Information). To calculate the spreading of the plume in the micromodel, the diffusivity of glutamate (molecular weight *M* = 147) in the artificial water was derived from the Stokes–Einstein equation,^[^ ^81^ ^]^ which provides *D*_glu_ = 608 µm^2^ s^−1^ for our experimental conditions (dynamic viscosity = 1.0 × 10^−3^ kg m^−1^ s^−1^, salinity = 36 g kg^−1^, temperature = 23 °C).

To quantify bacterial chemotaxis, the microchannels were imaged on an inverted microscope (Nikon Eclipse Ti‐E) in phase contrast, using a 10× (N.A. 0.30) objective. Videos were captured at a frame rate of 30 fps for 100 s with an sCMOS camera (Hamamatsu) controlled through Nikon Elements software. It began to acquire videos 10 s before the uncaging of glutamate to initially capture trajectories of bacteria that were not performing chemotaxis. The bacterial trajectories with custom MATLAB (MathWorks) scripts was reconstructed. From the reconstructed bacterial trajectories, accumulation profiles of bacteria within the plume were released from the pillar after the uncaging was extracted. The mean swimming speed, *v* = 46.5 µm s^−1^, of the bacteria was taken from previous work in which the cells were cultured and imaged under the same experimental conditions.^[^ ^52^ ^]^

## Conflict of Interest

The authors declare no conflict of interest.

## Supporting information

Supporting Information

## Data Availability

The data that support the findings of this study are available from the corresponding author upon reasonable request.
