# Supplementary material for: Multiscale Porosity Microfluidics to Study Bacterial Transport in Heterogeneous Chemical Landscapes
Source: Adv Sci (Weinh). 2024 Mar 6;11(20):2310121. doi: 10.1002/advs.202310121 (PMC11132056; doi:10.1002/advs.202310121)
Supplement: Supplementary file 1 — Supporting Information [file ADVS-11-2310121-s001.pdf]

ADVANCED SCIENCE
Open Access

Supporting Information

for *Adv. Sci.*, DOI 10.1002/advs.202310121

Multiscale Porosity Microfluidics to Study Bacterial Transport in Heterogeneous Chemical Landscapes

M. Mehdi Salek, Francesco Carrara, Jiande Zhou, Roman Stocker and Joaquin Jimenez-Martinez**

Supporting Information

Multiscale Porosity Microfluidics to Study Bacterial Transport in Heterogeneous Chemical Landscapes

M. Mehdi Salek[1,2,*], Francesco Carrara[2], Jiande Zhou[2,3],
Roman Stocker[2], Joaquin Jimenez-Martinez[2,4,*]

[1] School of Engineering, Massachusetts Institute of Technology, Cambridge, MA, USA

[2] Institute of Environmental Engineering, Department of Civil, Environmental and Geomatic Engineering, ETH Zurich, Zurich, Switzerland

[3] Microsystems Laboratory, Institute of Microengineering, School of Engineering, EPFL, Lausanne, Switzerland

[4] Department of Water Resources and Drinking Water, Eawag, Dubendorf, Switzerland

*Corresponding authors: msalek@mit.edu; joaquin.jimenez@eawag.ch

Supplementary Text

Hydrogel pillars microstructure. The polyacrylamide (PAAm) hydrogel pillars were created using an established *in situ* photopolymerization protocol (Nissan et al., 2021, de Schaetzen et al., 2022). The polymer solution was composed of 10 mL 20 wt% acrylamide (T) and 1 wt% N,N–methylenebisacrylamide as a cross-linker (C) plus 200 mg Irgacure for the photopolymerization. The microfluidic channel was filled with the hydrogel polymer solution, and then the gel was locally cured at the desired locations within the microfluidic chip by UV photo-polymerization. Polymerization to form the pillars was achieved by exposing the polymer solution to a UV beam according to standard procedure for in situ polymerization (Calio et al., 2015) by the use of a 20× objective (Nikon CFI S Plan Fluor ELWD 20XC) on a Nikon Ti microscope with a DAPI cube (EX: 350/50 nm; Chroma 49025) connected to a metal halide fluorescent light source (Prior Lumen 200) at 100% power, while simultaneously chemically bonding the walls to the top and bottom PDMS layers at 293 °K (Yuk et al., 2016; de Schaetzen et al., 2022). The exposure time was set to 15 s for all hydrogel pillars in the micromodels.

The final hydrogel permeability to amino-acids or other small proteins can be tuned by changing the concentration of the polymer solution (in our case T = 21%) and the ratio of cross-linker (in our case C = 4.75%), as described in seminal contributions (see for example Fawcett and Morris, 1966; Calvet et al., 2004) as well as in more recent contributions (see e.g., Denisin and Pruitt, 2016). Although no single standard method for nano- and microscale characterization of these hydrogels exists, we refer the reader to the work by Denisin and Pruitt (2016) (see Figure 2 in the main manuscript) on the mechanical characterization of polyacrylamide hydrogels as a function of different values of acrylamide T (in the range 5–25%) and cross-linker C (2–10%) contents. From their analysis, based on a combination of Atomic Force Microscopy (AFM) and Surface field-emission *(FE)* scanning electron microscopy *(SEM) FESEM* (see Figure 3 in the main manuscript) the authors claim that "*Decreasing stiffness at high cross-linker concentrations can be explained by a transition in the structure of the polyacrylamide gel network from an ideal to a clustered gel. Ideal hydrogels exhibit maximal elasticity because each tetrafunctional bis-acrylamide molecule is connected to four of the nearest neighboring acrylamide groups and spaced following a Poisson distribution to ensure maximal elastically effective chains.*"

Based on the combined evidence from the experimental work of Denisin and Pruitt (2016) and on the characterization of PAAm hydrogels provided by Calvet et al., 2004), we can conclude that the PAAm hydrogel used in our study (T = 21%, C = 4.75%) is behaving as an ideal hydrogel with pore scale at the nanometer scale, and extremely close to its maximum elasticity (see Figures 1, 2 reported from Denisin and Pruitt, 2016).

Triple porosity mimicking soil microenvironments. Soil microorganisms (mainly bacteria) live in periodically interconnected communities closely associated with soil aggregates. To improve our understanding of the biogeochemical processes in soils, it is necessary to understand the micrometer-scale interactions between soil particles and microbes. Our device allows us to study soil ecosystems at a scale relevant to individual bacteria. The rationale behind using this geometry is the conceptualization of soil macroaggregates and microaggregates, i.e., the hierarchical, self-organization of soils (Yudina and Kuzyakov,

2023). Microaggregates (250-500 μm), which bind a soil organic particle, are embedded in macroaggregates (up to 2 mm), which control oxygen diffusion and fluid flow. Conceptually speaking, our micromodel would be a macroaggregate, and the hydrogel pillars surrounded by the corona of PDMS small pillars would be the microaggregates. Once the nutrient is punctually uncaged, the hydrogel pillar would correspond to the particulate organic matter (POM). While high fluid flow velocities occur between macroaggregates, lower fluid flow velocities occur within them, i.e., between microaggregates. Flow through and around the macroaggregates allows the dispersion of nutrients (e.g., dissolved organic carbon) and of microbiology (e.g., bacteria, viruses).

Further, in our experiments, we used as a caged chemoattractant 4-methoxy-7-nitroindolinyl-(MNI)-caged-L-glutamate. MNI-caged glutamate is a version of the amino acid glutamate, a chemoattractant for many bacteria. Similar compounds, such as poly-γ-glutamic acid (γ-PGA), a polymer, are also responsible for the soil aggregates stability and improve soil water holding capacity and plant growth (Liu et al., 2023). This aspect further frames our work in the context of soil processes.

On geometry and configuration of the porosity micromodels. By performing a similar analysis as the one presented in the main manuscript, we tested how the chemotactic response of a bacterial population depends on important porosity parameters. Specifically, we designed two additional micromodels: in the first, we used the same geometrical configuration of the triple-porosity micromodel presented in the main manuscript, except for the removal of the corona of PDMS small pillars surrounding the hydrogel pillar. This allowed us to test the effect of the nested geometry of the microaggregate on the bacterial response. In the second, we removed the corona of PDMS small pillars and we increased by 56 % the size of the hydrogel pillar to a radius r = 390 μm (original pillar size r = 250 μm). This allowed us to test the effect of the POM size of the microaggregate on the microbial chemotaxis response as a function of time from the nutrient injection in the system (Figure S3).

We found that the bacterial accumulation around the central hydrogel pillar in the dual-porosity micromodels was reduced by more than 50% (Figures S4a,c) when compared to the triple-porosity micromodel which is used in the main manuscript (Figure 5e), at comparable mean flow speed and glutamate concentrations (see Figure S1). The greater retention of bacteria sustained over longer times observed in the triple-porosity micromodel is due to a combination of lower flow velocities within the PDMS corona region and chemoattractant concentrations and therefore chemical gradients that are sustained at higher levels and over longer timescales. At the same time, the larger hydrogel pillar in the dual-porosity maintained a larger number of bacteria for a longer period of time (Figure S4c) compared to the small hydrogel pillar (Figure S4a), which has the same dimension as the one used in the main manuscript (see also Figure 2). The lower accumulation is induced by a stronger washout effect in the region downstream of the hydrogel pillar, where the screening of the flow velocity field provided by the pillar is reduced compared to the larger pillar (Figures S4b,d).

Supplementary Figures

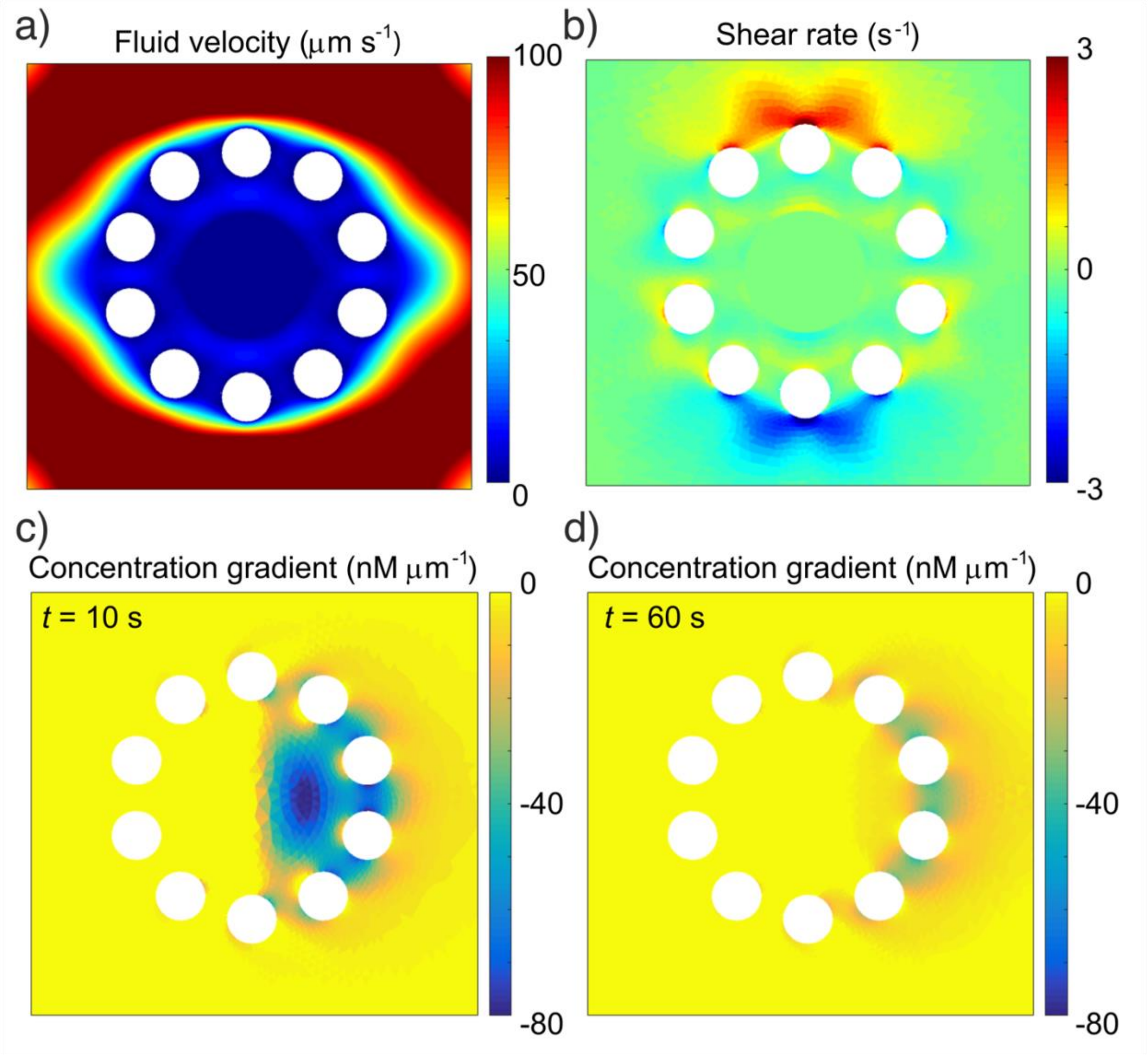

Figure S1. Hydrodynamic conditions and concentration gradients in the region of one pillar cluster in the triple-porosity micromodel. Spatial distribution of the flow velocity **(a)**, shear rate **(b)**, and the chemoattractant concentration gradient at time t = 10 s **(c)** and t = 60 s **(d)** after a nutrient pulse. Continuous estimates of the spatial distribution of the concentration gradient over time like these were used to calculate the spatial averages shown in Figure 5i.

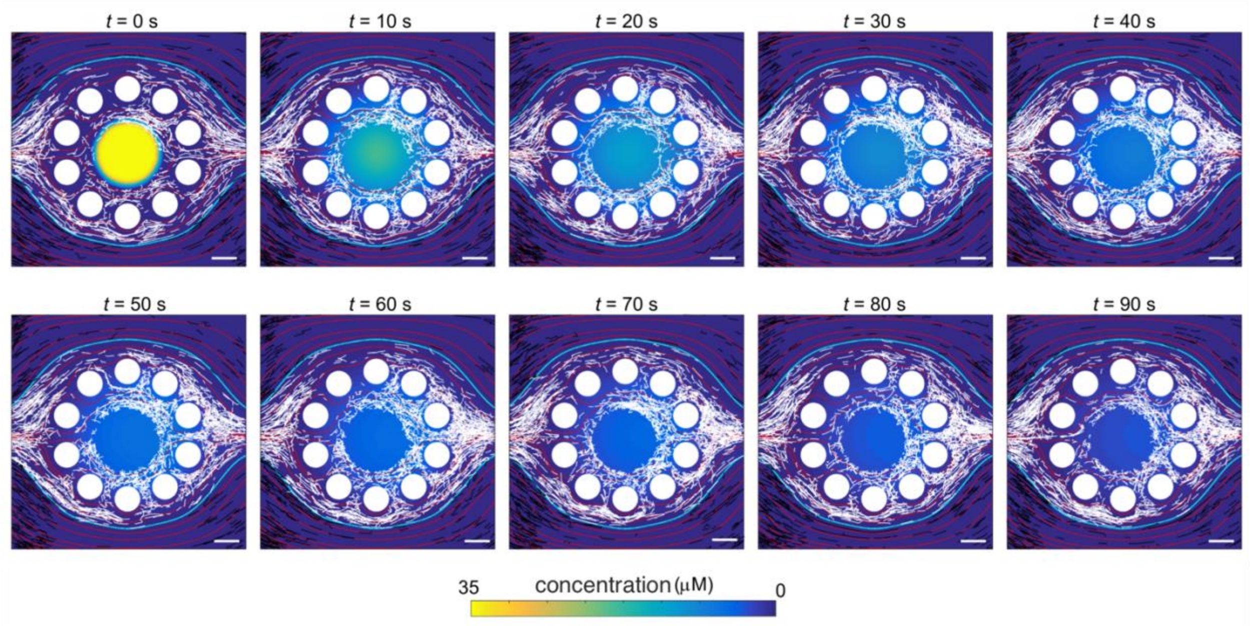

Figure S2. Temporal dynamics of microbial chemotaxis under flow conditions in response to a nutrient hotspot within a triple porosity micromodel. Bacterial trajectories over 10 s (black and gray tracks; frame rate 30 fps) of a population of *Vibrio ordalii* are superimposed over the streamlines (red) and the nutrient concentration field (color-coded) around one pillar cluster. Panels show a time series (t = 0 s to 90 s) after the nutrient began to diffuse out of the central hydrogel pillar (at t = 0 s, yellow circle). The glutamate nutrient pulse generated by photolysis had an initial cross-section equivalent to the central hydrogel pillar (250 μm). The uncaged glutamate was made instantly available to the bacteria at an initial concentration of 35 μM. Cyan lines indicate the contour in the flow field where flow velocity u = 100 μm s^{-1}. Bacterial trajectories are color-coded according to their position in the flow field: high flow (u > 100 μm s^{-1}), black; medium flow (10 μm s^{-1} < u < 100 μm s^{-1}), gray. Scale bar is 100 μm.

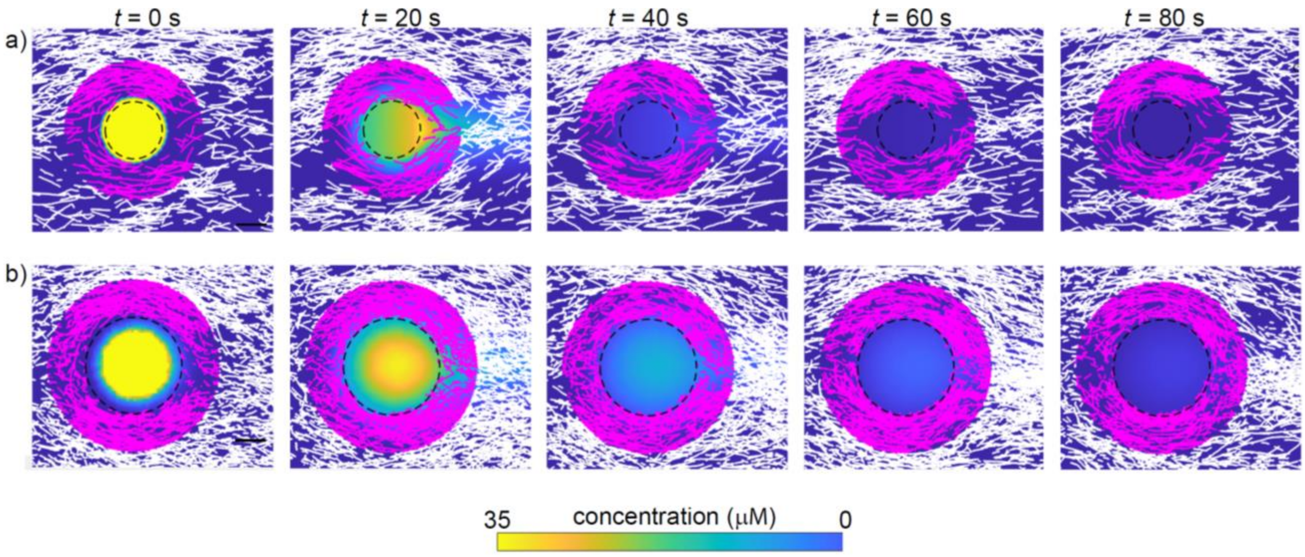

Figure S3. Microbial chemotaxis under flow conditions in response to nutrient hotspots within double-porosity micromodels. Bacterial trajectories over a 10 s period of a population of *Vibrio ordalii* superimposed over the nutrient concentration field (color-coded), for the periods *t* = -5–5 s, *t* = 15–25 s, *t* = 35–45 s, *t* = 55–65 s and *t* = 75–85 s after the uncaging of the glutamate (initial glutamate concentration C = 35 μM) in the central hydrogel pillar (black dashed circle) with diameter 250 μm (**a**) and 390 μm (**b**). Exposure to a focused LED beam (diameter 250 μm) uncages glutamate through photolysis. There is a constant flow through the micromodel of a solution containing caged glutamate and bacteria (fluid flow is from left to right). For both micromodels, the glutamate nutrient pulse had an initial cross-section of 250 μm. Bacterial trajectories are color-coded according to their radial distance from the central pillar: magenta color represents an annulus of radial thickness of 150 μm around the hydrogel pillar. For comparison with the triple-porosity micromodel, magenta trajectories virtually correspond to the region within the PDMS crown (subdomain Γ_2). Scale bar is 100 μm.

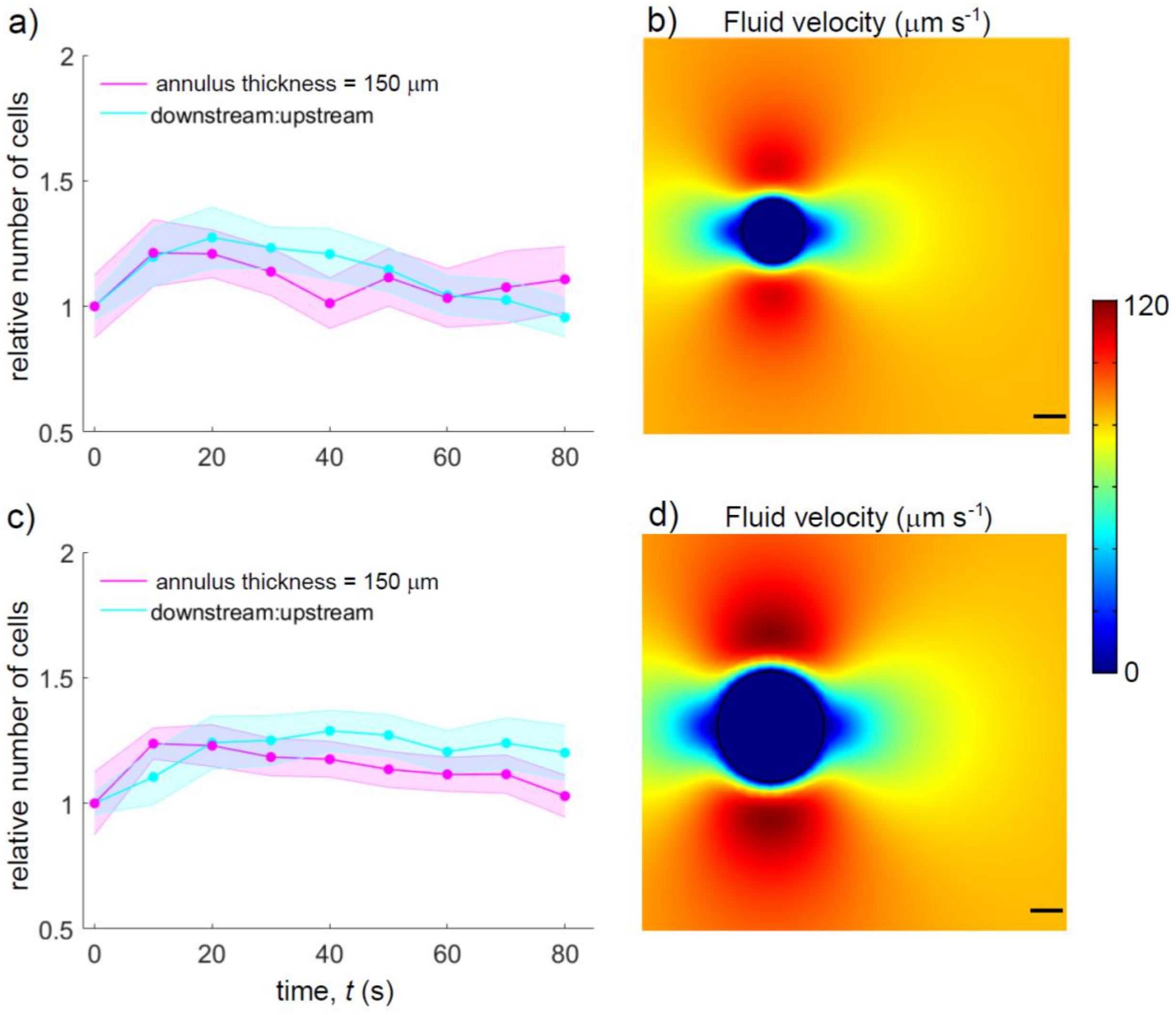

Figure S4. The relative number of bacteria responding to a chemical pulse depends of the geometry of the double-porosity micromodel. For both sizes of the hydrogel pillar, 300 μm **(a)**, and 390 μm **(c)**, the concentration of bacteria quickly increases after a nutrient pulse (t = 0 s to t = 20 s) within the central annular region virtually corresponding to the region within the PDMS crown (subdomain Γ_2, not present here) and downstream of the pulse. However, only for the larger hydrogel pillar diameter (390 μm), the bacterial accumulation can be sustained at longer times (> t = 60 s). Magenta curve: number of bacteria over time within the annular region around the hydrogel pillar (with thickness 150 μm) relative to the number of bacteria at time t = 0 s. Cyan curve: number of bacteria downstream (right-hand side) relative to the number of bacteria upstream (left-hand side) within the flow region with u < 100 μm s^{-1} (see panels **b** and **d** for the color-coded flow velocity fields corresponding to the small and large pillar micromodels, scale bar is 100 μm). Shaded regions in panels **a** and **c** show the ± s.d. of the bacterial trajectories over the corresponding time interval.

Supplementary References

Calio, A., Leng, J., Decock, J., De Stefano, L. and Salmon, J.B., 2015. Microscopy assisted fabrication of a hydrogel-based microfluidic filter. *Journal of the European Optical Society-Rapid Publications*, *10*.

Calvet, D., Wong, J.Y. and Giasson, S., 2004. Rheological monitoring of polyacrylamide gelation: Importance of cross-link density and temperature. *Macromolecules*, *37*(20), pp.7762-7771.

Denisin, A.K. and Pruitt, B.L., 2016. Tuning the range of polyacrylamide gel stiffness for mechanobiology applications. *ACS Applied Materials & Interfaces*, *8*(34), pp.21893-21902.

de Schaetzen, F., Fan, M., Alcolombri, U., Peaudecerf, F.J., Drissner, D., Loessner, M.J., Stocker, R. and Schuppler, M., 2022. Random encounters and amoeba locomotion drive the predation of Listeria monocytogenes by Acanthamoeba castellanii. *Proceedings of the National Academy of Sciences*, *119*(32), p.e2122659119.

Fawcett, J.S. and Morris, C.J.O.R., 1966. Molecular-sieve chromatography of proteins on granulated polyacrylamide gels. *Separation Science*, *1*(1), pp.9-26.

Nissan, A., Alcolombri, U., de Schaetzen, F., Berkowitz, B. and Jimenez-Martinez, J., 2020. Reactive transport with fluid–solid interactions in dual-porosity media. *ACS ES&T Water*, *1*(2), pp.259-268.

Yuk, H., Zhang, T., Lin, S., Parada, G.A. and Zhao, X., 2016. Tough bonding of hydrogels to diverse non-porous surfaces. *Nature Materials*, *15*(2), pp.190-196.

Liu, L., Shi, W., Liang, J., Yuan, Y. and Hao, Q., 2023. Soil macropores characteristic and aggregate stability with poly-γ-glutamic acid amendment under wetting-drying cycles. *European Journal of Soil Science*, p.e13361.

Wilpiszeski, R.L., Aufrecht, J.A., Retterer, S.T., Sullivan, M.B., Graham, D.E., Pierce, E.M., Zablocki, O.D., Palumbo, A.V. and Elias, D.A., 2019. Soil aggregate microbial communities: towards understanding microbiome interactions at biologically relevant scales. *Applied and Environmental Microbiology, 85*(14), pp.e00324-19.

Yudina, A. and Kuzyakov, Y., 2023. Dual nature of soil structure: The unity of aggregates and pores. *Geoderma*, *434*, p.116478.
